# Investigations into the growth and formation of biofilm by *Leptospirabiflexa* at temperatures encountered during infection

**DOI:** 10.1016/j.bioflm.2024.100243

**Published:** 2024-12-10

**Authors:** Priscyla dos Santos Ribeiro, Judith Stasko, Adrienne Shircliff, Luis Guilherme Fernandes, Ellie J. Putz, Claire Andreasen, Vasco Azevedo, Paula Ristow, Jarlath E. Nally

**Affiliations:** a Federal University of Minas Gerais, Belo Horizonte, Brazil; b Federal University of Bahia, National Institute of Science and Technology in Interdisciplinary and Transdisciplinary Studies in Ecology and Evolution, Salvador, Brazil; c Infectious Bacterial Diseases Research Unit, USDA Agriculture Research Service, National Animal Disease Center, Ames, IA, USA; d Department of Veterinary Pathology, College of Veterinary Medicine, Ames, IA, USA

**Keywords:** Proteins, DNA, Biofilm matrix, Leptospirosis, *Leptospira* spp

## Abstract

The genus *Leptospira* comprises unique atypical spirochete bacteria that includes the etiological agent of leptospirosis, a globally important zoonosis. Biofilms are microecosystems composed of microorganisms embedded in a self-produced matrix that offers protection against hostile factors. Leptospires form biofilms *in vitro, in situ* in rice fields and unsanitary urban areas, and *in vivo* while colonizing rodent kidneys. The complex three-dimensional biofilm matrix includes secreted polymeric substances such as proteins, extracellular DNA (eDNA), and saccharides. The genus *Leptospira* comprises pathogenic and saprophytic species with the saprophytic *L. biflexa* being commonly used as a model organism for the genus. In this study, the growth and formation of biofilms by *L. biflexa* was investigated not just at 29 °C, but at 37 °C/5 % CO_2_, a temperature similar to that encountered during host infection. Planktonic free-living *L. biflexa* grow in HAN media at both 29 °C and 37 °C/5 % CO_2,_ but cells grown at 37 °C/5 % CO_2_ are longer (18.52 μm ± 3.39) compared to those at 29 °C (13.93 μm ± 2.84). Biofilms formed at 37 °C/5 % CO_2_ had more biomass compared to 29 °C, as determined by crystal violet staining. Confocal microscopy determined that the protein content within the biofilm matrix was more prominent than double-stranded DNA, and featured a distinct layer attached to the solid substrate. Additionally, the model enabled effective protein extraction for proteomic comparison across different biofilm phenotypes. Results highlight an important role for proteins in biofilm matrix structure by leptospires and the identification of their specific protein components holds promise for strategies to mitigate biofilm formation.

## 1 Introduction

Biofilms are aggregates of autotrophic and/or heterotrophic microorganisms embedded in a self-produced exopolymeric matrix [[1], [2], [3]]. Prokaryotes may adopt a biofilm lifestyle as it offers protection against unfavorable environmental factors, including antibiotics, disinfectants, and the host immune system [4,5]. Bacterial biofilms result in significant economic losses on a global scale, impacting human and animal health, industry, agriculture, and water quality [6].

The development of a biofilm *in vitro* is a step-by-step process that involves: 1) cell-to-cell and cell-to-substrate adhesion, 2) cellular growth and division leading to surface colonization, 3) biofilm maturation with formation of multiple layers and matrix production, and 4) detachment and dispersion of biofilm fragments or planktonic cells [7]. Biofilms have complex three-dimensional architectures: after the initial phase, cells produce an exopolymeric matrix consisting of different types of biopolymers known as extracellular polymeric substances (EPS), comprising proteins, extracellular DNA (eDNA), saccharides, and water [5].

Pathogenic and non-pathogenic *Leptospira* spp. form biofilms *in vitro* when cultivated at 28–30^o^C with Ellinghausen-McCullough-Johnson-Harris (EMJH) media [[8], [9], [10], [11], [12], [13]] and T80/40/LH [14]. The maturation time of those biofilms varies between species, ranging from two days for the saprophytic species *Leptospira biflexa* and *L. limi* [8,10,13] and up to 21 days for pathogenic *L. interrogans* [9,14]. Additionally, pathogenic leptospires form biofilms *in vivo* in kidneys of naturally infected rats acting as reservoir hosts of infection in communities endemic for leptospirosis [15], and *in situ* in rice fields in India [12]. A diversity of non-pathogenic *Leptospira* species were isolated from naturally forming biofilms in an urban community with precarious sanitary conditions [10].

To further understand biofilms formed by leptospires, the non-pathogenic species *L. biflexa* has been used as a model organism. *L. biflexa* grows relatively fast, is non-pathogenic, and easily manipulated. Additionally, *L. biflexa* shares some phenotypic characteristics with pathogenic leptospires, such as the ability to form biofilms adhered to glass surfaces and floating biofilms [8]. Furthermore, they exhibit genomic similarities in genes possibly related to biofilm formation [8,11], making *L. biflexa* a valid model for studying biofilm formation mechanisms.

The complete transcriptome of *L. biflexa* in mature and late-stage biofilms compared to planktonic cells at the same time points demonstrated that leptospires in biofilms undergo changes in gene expression related to growth, replication, outer membrane proteins (OMP), and regulators [11]. OMPs are proteins located in the outer membrane of Gram-negative bacteria and that can function in the transport of molecules to the cell external environment [16]. Differences in the expression of OMPs by bacteria in biofilms are widely reported, with their expression often being upregulated in biofilm-associated cells [11,[17], [18], [19]]. Among the growth-related genes differentially expressed in the transcriptome of *L. biflexa* in biofilms, a notable example is the *carD* family transcriptional regulator, which exhibited differential expression in biofilms compared to planktonic cells [11]. In *Mycobacterium smegmatis*, *carD* was upregulated in response to stress conditions induced during nutrient deprivation [20]. Mutation of the phosphodiesterase encoding genes (*pdeA*::Km and *pdeB*::Km) in pathogenic *L*. *interrogans* resulted in mutants characterized as biofilm super-producers. Phosphodiesterase degrades the signaling molecule bis-(3′–5′)-cyclic dimeric guanosine monophosphate (c-di-GMP) [9]. These mutants showed a higher level of cellular activity in mature biofilms after exposure to conditions that mimic environmental stresses (pH, temperature, NaCl concentration, tetracycline, and ultraviolet radiation) compared to wild-type strains, as well as mutants with reduced biofilm production (*dgcA*::Km and *akrA*::Km, diguanylate cyclase and the aldo-keto reductase encoding genes, respectively) [9]. *In silico* analyses of *L. biflexa* led to the identification of 40 proteins potentially related to c-di-GMP turnover and signaling. Furthermore, metabolic predictions showed that c-di-GMP signaling potentially interacts with other signaling or regulatory systems, such as cAMP and CsrA systems, as well as endoflagella assembly regulation, in *L. biflexa* [21].

In the present work, the growth of, and biofilm formation by, the non-pathogenic model species *L. biflexa* is described in HAN media at different growth conditions (29 °C versus 37 °C/5 % CO_2_) that emulate biofilm formation at optimal laboratory and mammalian host-like temperatures, respectively. Using biofilm confocal microscopy and electron microscopy, the composition of the biofilm matrix and its structures were investigated. Furthermore, proteomic analysis of biofilms formed by *L*. *biflexa* at 29 °C indicated differential protein expression compared to those formed at 37 °C/5 % CO_2_.

## 2 Methods

**Planktonic *Leptospira.****Leptospira biflexa* serovar Patoc strain Patoc 1 (ATCC 23582) and *Leptospira interrogans* serogroup Canicola strain LAD1 were propagated under static conditions, at 29 °C and at 37 °C/5 % CO_2_, by inoculating 10 mL of HAN media [22] in a 50 mL conical plastic tube with 1 mL containing 10^6^ leptospires. Growth curves of *L. biflexa* planktonic cells were performed by counting cells using dark field microscopy at days 1, 2, 3, 4 and 7 post-inoculation [23]. Six independent experiments were performed, and the average and standard error were calculated using Excel.

**Fig. 1:**
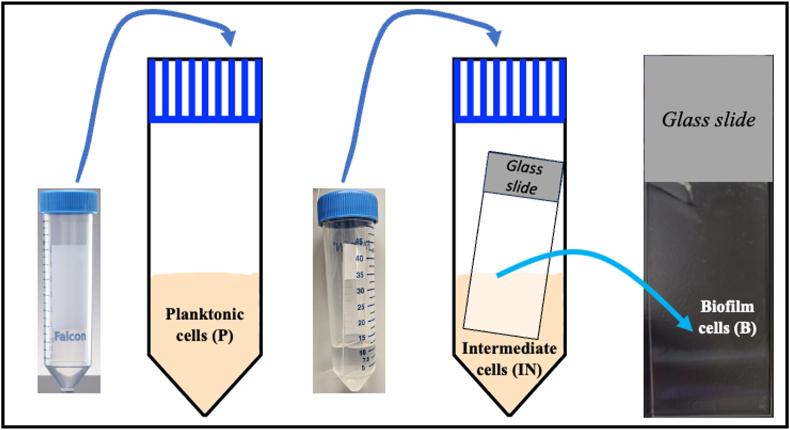
Schematic of the samples analyzed in this study. Planktonic cells (P) = free-living cells; Intermediate cells (IN) = free-living cells, cells detached from the biofilm, and cells that can attach to the biofilms; and Biofilm (B) = cells adhered to the glass slide support.

**Biofilms of *Leptospira***. Biofilms were cultivated adhered to a sterile glass slide (Superfrost™ slides, epredia) inserted in a 50 mL conical plastic tube (Falcon) [10] by inoculating 10 mL of HAN media with 1 mL containing 5x10^6^ leptospires (Fig. 1) [8]. Intermediate cells (IN) were obtained from the conical plastic tubes (Falcon) used for biofilm formation and include free-living cells, cells detached from the biofilm, and cells that can attach to the biofilms.

**Crystal violet staining and indirect biofilm quantification.** Biofilm formation was assessed by crystal violet (CV) staining on days 4, 5, 6, 7, 8, 9, 10, 11 and 14 post-inoculation to evaluate biofilm formation by *L. biflexa* in HAN media and to determine the maturation phase under these conditions. The glass slide with adhered biofilm was gently removed from the conical tube and rinsed delicately with distilled sterile water. The slide was then dried under a heat lamp (SATCO R40 Infrared reflector 250W) for 20 min, fixed with 2 % sodium acetate solution (g/v) for 20 min and dried again. Slides were then stained with 0.1 % CV solution (g/v) for 20 min, followed by gentle washing twice with distilled sterile water. The remaining CV was solubilized in 30 % (v/v) acetic acid solution, which was read in a spectrophotometer with absorbance at 600 nm [10]. Glass slides inserted into conical tubes containing HAN media without leptospires, and taken at the same time points, were used as negative controls.

**Confocal Laser Scanning Microscopy (CLSM).** Biofilm structure and composition at different stages of biofilm formation was assessed by CLSM analysis at days 4, 7, 8, 9, 10, 11 and 14 post-inoculation. For that the glass slide with adhered biofilm was gently removed from the conical tube and rinsed delicately with distilled sterile water. Biofilms were then fixed with 2 % paraformaldehyde (PFA) in Phosphate Buffered Saline (PBS) (Sigma) for at least 24 h until further analysis. Biofilms were stained for 30 min with 200 μL of FilmTracer Sypro Ruby Biofilm Matrix Stain (Invitrogen™), washed x 4 with distilled sterile water, covered with 30 μL of antifade mounting medium with DAPI (VECTASHIELD®), and cover slipped.

For CLSM, an EclipseTi microscope model was used for visualization. NIS-Elements imaging software (Nikon Instruments, Melville, NY) was used to acquire 2 and 3-D images. The COMSTAT2 software [24,25] was used to analyze the biomass of DAPI (double stranded DNA; green) and Sypro Ruby (biofilm matrix proteins; red) channels [25]. Biomass, as measured by COMSTAT software, is quantified as the volume of all voxels (pixels) with levels above the threshold divided by the surface area [24]. The 40X oil objective with a refractive index of 1.515 was used. DAPI fluorescence signal had an emission wavelength of 450 nm and excitation wavelength of 403 nm; Sypro Ruby had an emission wavelength of 595 nm and excitation wavelength of 488.1 nm.

**Transmission Electron Microscopy (TEM) negative staining.** Planktonic and biofilm leptospires were grown as described and samples promptly prepared for TEM to ensure preservation of cells and biofilm structures. For the measurement of planktonic cells, 10 μL of a mid-late log phase culture (∼2 x 10^9^ leptospires/mL) was used. To this volume, 10 μL of 2.0 % phosphotungstic acid (PTA, pH 7, Electron Microscopy Sciences) was added and incubated for 3 min. The total volume was then placed on a copper 200 mesh formvar carbon film grid (Electron Microscopy Sciences, Hatfield, PA). The lengths of ten cells from each condition were measured, and the average and median lengths were calculated using Excel. The experiment was repeated twice, independently. For characterization of biofilm ultrastructure, biofilms were grown as described and the glass slide with adhered biofilm was delicately washed with distilled sterile water. The 200 mesh formvar coated grid was used to scrape off a portion of the biofilm from the slide and placed so that the grid was facing up for incubation with 2.0 % PTA (pH 7.0, Electron Microscopy Sciences) for 3 min or with 0.2 % ruthenium red (RR, Polysciences, Warrington, PA) for 5 min. The grids were then visualized on a ThermoFisher FEI Tecnai G2 BioTWIN electron microscope (FEI Co., Hillsboro, OR) and images were taken with Nanosprint12 camera (AMT Corp., Woburn, MA).

**Data analysis.** Statistical analyses were performed for CV samples at days 4, 5, 6, 7, 8, 9, 10, 11 and 14, and for CLSM samples on days 4, 7, 8, and 9. These experiments were performed three times independently, and for CLSM analysis, in each experiment, three fields were randomly imaged. A two-way ANOVA test followed by Tukey's test for pairwise comparisons were performed using RStudio (Version 2024.04.1). Data are represented as boxplot graphs where dots represent minimum, maximum, and outlier data; the central box represents the interquartile range and the line inside the box shows the median value. For analysis of cell measurements by TEM, two independent experiments were performed using 10 different cells per condition, and the average, standard deviation, and median were calculated using Excel.

**1-D gel electrophoresis and immunoblotting.** Planktonic (P), intermediate (IN) and biofilm (B) cells were grown as described (Fig. 1). Planktonic cells were grown until mid-late log phase (∼2 x 10^9^ leptospires/mL). Cells were harvested by centrifugation (10,000×*g*, 4 °C, 30 min), washed twice with ice-cold TE buffer, and kept at −20 °C until further analysis. Biofilms were grown until the maturation phase (7 days), delicately washed with distilled sterile water, and kept at −80 °C until further analysis. Biofilms were scraped from the glass slide using a sterile scalpel and resuspended in 1.5 mL of ice-cold sterile distilled water. Cells were harvested by centrifugation (15,000×*g*, 4 °C, 15 min) and kept at −20 °C until further analysis. Intermediate cells were processed as described for planktonic cells.

Planktonic and intermediate cell pellets were resuspended in 400 μL SDS-PAGE solubilization buffer, boiled for 10 min, and the equivalent of 2 x 10^8^ leptospires was loaded per lane. Biofilm biomass was resuspended in 50 μL SDS-PAGE solubilization buffer, boiled for 10 min, and 5 μL was loaded per lane. The gels were processed for one-dimensional (1-D) SDS-PAGE on 12 % acrylamide gels (BioRad, CA, USA) following the manufacturer's guidelines. Proteins were visualized by staining with Sypro Ruby (Invitrogen, CA, USA) [26].

Samples were subjected to semi-dry transfer using an Amersham TE77 PWR apparatus onto PVDF membranes (Bio-Rad). Then, membranes were blocked overnight at 4 °C with blocking buffer (Thermo Scientific), incubated with anti-GroEL (60 kDa) antisera at a 1:1000 dilution in blocking buffer. Subsequently, the membranes were washed three times for 5 min with PBS containing 0.1 % Tween 20 (PBS-T) and incubated with horseradish peroxidase-conjugated anti-rabbit immunoglobulin G diluted 1:2500 in blocking buffer. *L. interrogans* serogroup Canicola strain LAD1 was used as positive control. Detection of bound conjugates was achieved using Clarity Western ECL substrate (BioRad), and imaging performed using the Bio-Rad ChemiDoc MP system [26]. Representative images are provided.

## 3 Results

**Fig. 2:**
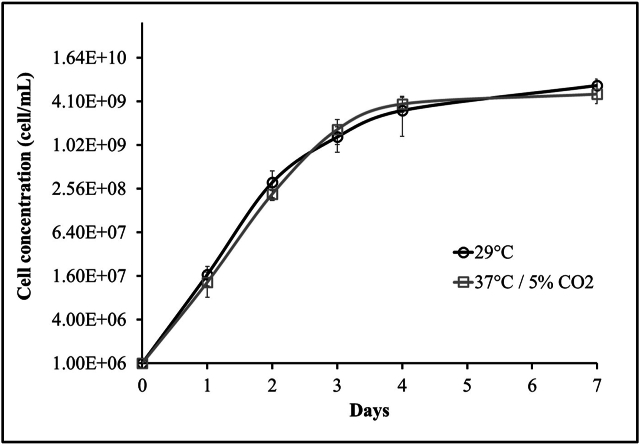
Growth curves of *Leptospira biflexa* serovar Patoc strain Patoc 1 planktonic cells grown in liquid HAN media at 29 °C versus 37 °C/5 % CO_2_. Data represents six independent replicas. Bars indicate standard errors.

**Fig. 3:**
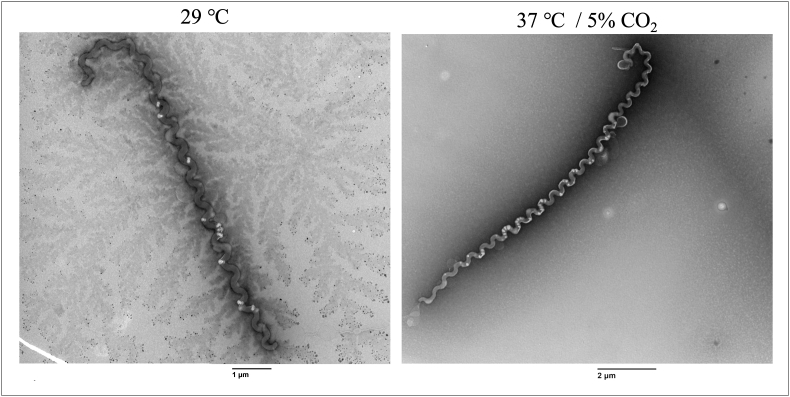
Representative images of cell length differences of *Leptospira biflexa* serovar Patoc strain Patoc 1 planktonic cells grown in liquid HAN media at 29 °C (original magnification x11,000) and at 37 °C/5 % CO_2_ (original magnification ×6800) by TEM.

***L. biflexa* planktonic cells grow in HAN media at different growth conditions.** Prior to assessing biofilm growth, it was established that planktonic free-living *L. biflexa* grow at similar growth rates when cultured in liquid HAN media at 29 °C or 37 °C/5 % CO_2_ (Fig. 2). Notably, differences in cell length of *L. biflexa* propagated under different conditions were observed by dark field microscopy. Hence, *L. biflexa* propagated in each condition were further compared by TEM and lengths of cells measured. *L. biflexa* propagated in HAN media at 29 °C were shorter (average: 13.93 μm ± 2.84; median: 12.93 μm) compared to *L. biflexa* propagated at 37 °C/5 % CO_2_ (average: 18.52 μm ± 3.39; median: 18.67 μm) (Fig. 3).

**Fig. 4:**
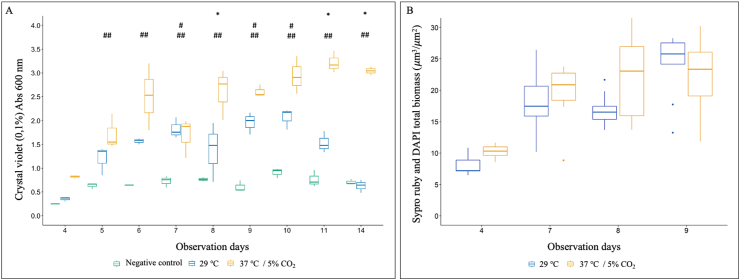
Quantification of *Leptospira biflexa* serovar Patoc strain Patoc 1 biofilm formation in liquid HAN media at 29 °C and at 37 °C/5 % CO_2_. **A –** Biomass of biofilms stained with 0.1 % (g/v) CV solution. Statistically significant differences (p < 0.05) in biomass was detected comparing ∗29 °C and 37 °C/5 % CO_2_), ^#^29 °C *versus* negative control, and ^##^37 °C/5 % CO_2_*versus* negative control. **B –** Biomass of biofilms by CLSM after staining with Sypro Ruby (biofilm matrix proteins) and DAPI (dsDNA, representing live and dead cells, and extracellular DNA). Three independent experiments were performed. Each independent experiment had three images analyzed.

**Biofilm formation by *Leptospira biflexa* is observed in HAN media at different growth conditions.** Biofilms of *L.* *biflexa* developed in HAN media at both 29 °C and 37 °C/5% CO_2_, forming a whitish film adherent to the glass slide with increased thickness at the air-liquid interface (indicated in Fig. 1). Biofilm biomass was quantified indirectly by staining the biofilm with 0.1 % crystal violet solution (Fig. 4A) and confirmed a maturation phase from day 4 and onwards at both temperatures. Biofilm biomass was higher at days 8, 11 and 14 in biofilms grown at 37 °C/5 % CO_2_ compared to 29 °C (p < 0.05), but statistically significant differences were not observed comparing biomasses at days 7, 9 and 10. Detachment started on day 11 at 29 °C and on day 14 at 37 °C/5 % CO_2_ (Fig. 4A).

**Fig. 5:**
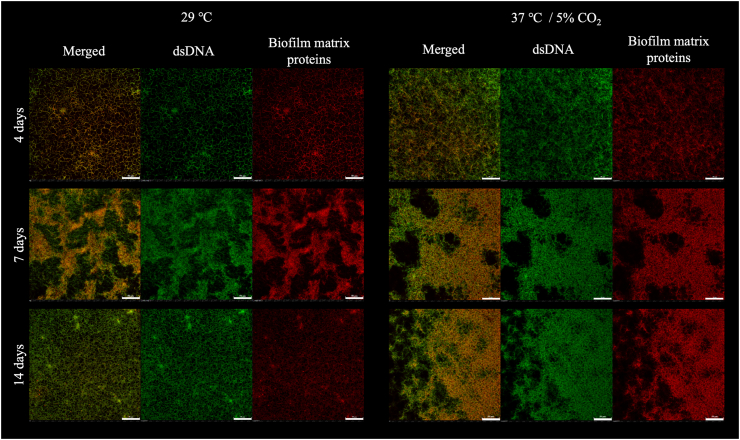
Structural characterization of *Leptospira biflexa* serovar Patoc strain Patoc 1 biofilm by CLSM. Biofilms were stained at days 4 (attachment phase), 7 (maturation phase), and 14 (detachment phase) with Sypro Ruby (red - for biofilm matrix proteins) and DAPI (green - dsDNA, representing live and dead cells and extracellular DNA). Representative 2D images of three independent experiments. 50 μm scale bar. (For interpretation of the references to colour in this figure legend, the reader is referred to the Web version of this article.)

To more accurately quantify biofilm biomass, confocal microscopy was performed with statistical analysis at days 4, 7, 8, and 9 as they represent the maturation phase of biofilm formation, as observed with the crystal violet assay. This selection also allowed us to investigate which days exhibited similar conditions at both temperatures, considering potential further analyses using proteomic techniques. For CLSM, the biofilms were stained with Sypro Ruby and DAPI that specifically stains biofilm matrix proteins and double-stranded DNA (dsDNA), respectively (Fig. 4, Fig. 5). DAPI staining of dsDNA detects both viable and non-viable cells due to its permeability through the cell membrane, as well as extracellular DNA (eDNA) [27]. Results further confirmed biofilm formation by *L. biflexa* propagated in HAN media at both 29 °C and 37 °C/5 % CO_2_ (Fig. 4, Fig. 5). To assess total biofilm biomass, confocal microscopy readings from Sypro Ruby and DAPI staining were combined; no statistical differences in the amount of biomass were observed between growth conditions (Fig. 4B). At day 7, the biomass was the same under both growth conditions (p > 0.05), whether measured by crystal violet or CLSM (Sypro Ruby plus DAPI) (Fig. 4A and B). Representative images of attachment, maturation and detachment phases by CLSM are provided (Fig. 5).

**Fig. 6:**
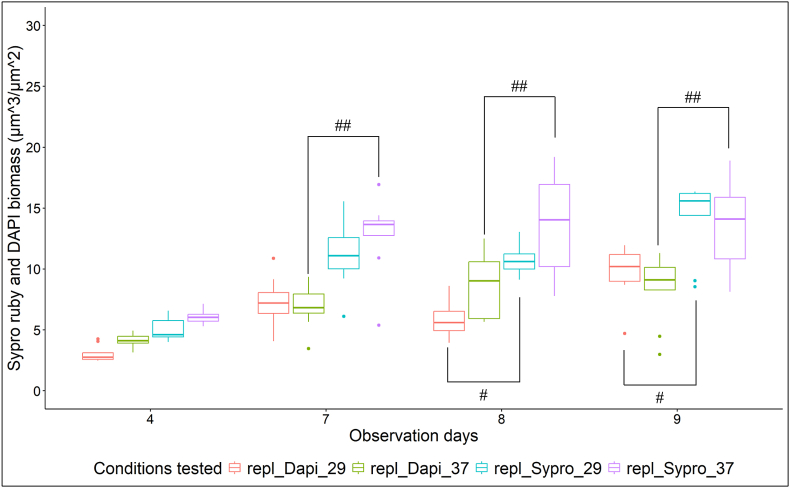
Quantification of protein and DNA in the biofilm matrix of *L. biflexa* at 29 °C and at 37 °C/5 % CO_2_. Biomass of biofilms were analyzed by CLSM and stained with Sypro Ruby (biofilm matrix proteins) and DAPI (dsDNA, representing live and dead cells and extracellular DNA). Three independent experiments were performed. Each independent experiment had three images analyzed. Statistically significant (p < 0.05) biomass was detected comparing ^#^ 29 °C _ Dapi *versus* Sypro Ruby and ^##^ 37 °C/5 % CO_2_ _ Dapi *versus* Sypro Ruby.

**Proteins are one of the major components of *L. biflexa* biofilm matrix.** Using confocal microscopy, individual analysis of Sypro Ruby and DAPI staining was performed to quantify differences in the biofilm matrix proteins and dsDNA biomasses, respectively, at 29 °C compared to 37 °C/5 % CO_2_ conditions (Fig. 6). The biomass detected by Sypro Ruby (biofilm matrix proteins) was significantly higher than that determined by DAPI (dsDNA) at 29 °C (days 8 and 9) and at 37 °C/5 % CO_2_ (days 7, 8, and 9) (p < 0.05) (Fig. 6).

**Fig. 7:**
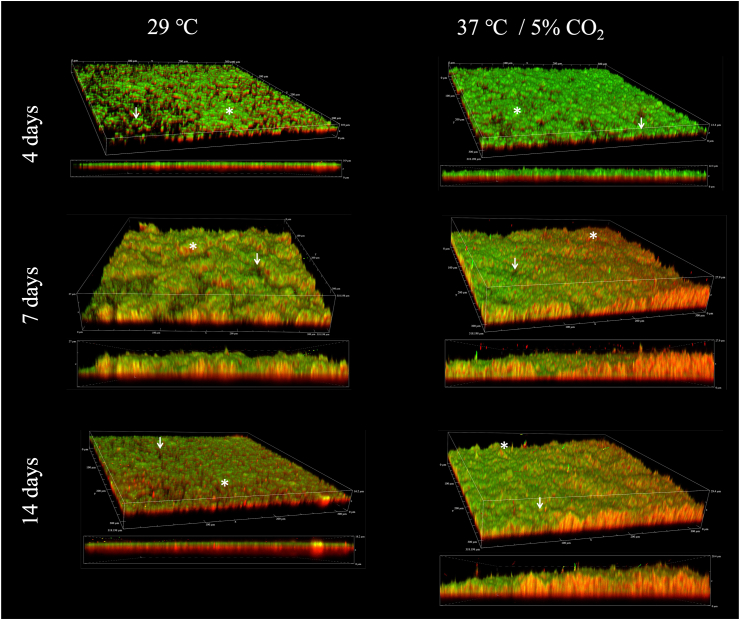
*Leptospira biflexa* biofilm matrix layers of protein (red, lower layer – stained with Sypro) and dsDNA (green, upper layer – stained with DAPI). Images obtained by CLSM. Cells were grown at 29 °C and at 37 °C/5 % CO_2_. Legend: white asterisks – biofilm hills (regions of dense vertical microbial growth or extracellular material deposition); white arrows – biofilm valleys (areas with relatively fewer biofilms' components). Representative images are shown. (For interpretation of the references to colour in this figure legend, the reader is referred to the Web version of this article.)

**Biofilms of *L.biflexa* have a layer of proteins adhered to the abiotic surface.** CLSM images demonstrated the three-dimensional (3D) structure of *L. biflexa* biofilm (Fig. 7). The analysis of XYZ-axis images showed an uneven structure of hills and valleys, where the “hills” are regions of dense vertical microbial growth or extracellular material deposition, whereas “valleys” are areas with relatively fewer biofilms’ components detected (Fig. 7). Additionally, proteins are observed between the dsDNA (cells and eDNA) in the biofilm matrix, providing structure and support to this phenotype. When the XY-axis images were analyzed, a distinctive and very clear layer of proteins was observed adhered to the glass slide surface (Fig. 7). This layer of protein was consistent in both growth conditions (29 °C and 37 °C/5 % CO_2_), as well as in all biofilm formation phases observed (Fig. 7).

**Fig. 8:**
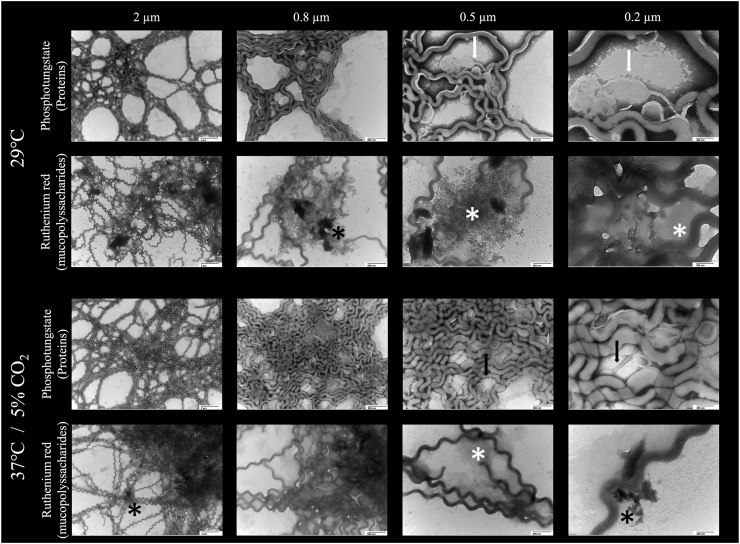
Ultrastructural characterization by negative staining TEM of *L. biflexa* biofilm during the maturation phase (7 days) propagated at 29 °C and at 37 °C/5 % CO_2_. Biofilms were stained with Ruthenium Red (acidic mucopolysaccharides) and Phosphotungstate (proteins). White arrow = vesicle-like structures; black arrow = flagella-like structures; white asterisk = less dense amorphous mucopolysaccharides; black asterisk = denser amorphous mucopolysaccharides. Original magnification for column 1 = x6,800, column 2 = x18,500, column 3 = x30,000 and column 4 = x68,000. From left to right, 2 μm, 0.8 μm, 0.5 μm, and 0.2 μm scale bars.

**Ultrastructural analysis of *L. biflexa* mature biofilm.** Transmission electron microscopy (TEM) negative staining was used to analyze the biofilm ultrastructure at the maturation stage (7 days). Biofilms were stained with phosphotungstic acid (PTA) which labels proteins, and ruthenium red (RR), which stains acidic mucopolysaccharides [28]. TEM images confirmed the presence of proteins and acidic mucopolysaccharides within the *L. biflexa* biofilm matrix cultured at both 29 °C and 37 °C/5 % CO_2_ (Fig. 8). Biofilms at 29 °C and 37 °C/5 % CO_2_ appeared as dense and intricate networks of microbial cells embedded within the extracellular matrix with void spaces. Leptospires were surrounded by proteins and mucopolysaccharides from the biofilm extracellular matrix. Protein staining using PTA detected vesicle-like structures (white arrow) and flagella-like structures (black arrow), while RR staining of mucopolysaccharides detected the presence of an amorphous matrix structure, which varied from less dense (white asterisk) to more dense structures (black asterisk) (Fig. 8).

**Fig. 9:**
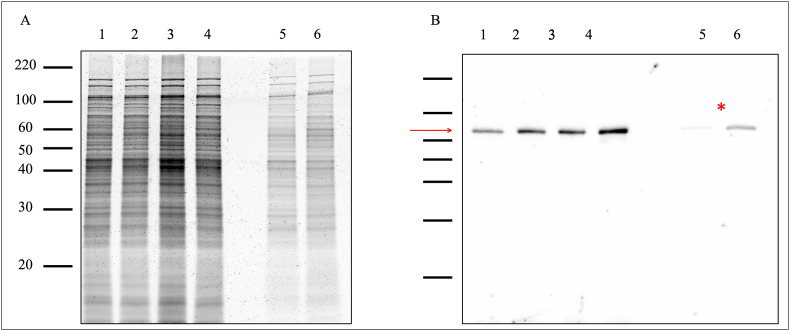
1-D gel electrophoresis of *L. biflexa* grown at 29 °C and 37 °C/5 % CO_2_. (A) Total protein profiles and (B) immunoblot to detect GroEL protein. (1) Planktonic at 29 °C; (2) Planktonic at 37 °C/5 % CO_2_; (3) Intermediate at 29 °C; (4) Intermediate at 37 °C/5 % CO_2_; (5) Biofilm at 29 °C; (6) Biofilm at 37 °C/5 % CO_2_. Asterisk indicates apparent decreased expression of GroEL in biofilms grown at 29 °C compared to 37 °C/5 % CO_2_. Red arrow indicates GroEL (60 KDa). (For interpretation of the references to colour in this figure legend, the reader is referred to the Web version of this article.)

**One-dimensional gel electrophoresis.** Biofilm formation by *L*. *biflexa* in HAN media resulted in the production of sufficient biofilm biomass that could be recovered for analysis by gel electrophoresis. Biofilm in the maturation phase (7 days), corresponding intermediate cells (7 days) (Fig. 1), and planktonic cells (mid-late log phase) were compared in each growth condition (29 °C and 37 °C/5 % CO_2_) for total protein content. To ensure consistency in the comparison between growth conditions, the equivalent of 2 x 10⁸ leptospires per lane was loaded for planktonic and intermediate cells. For biofilm samples, the protein concentration was normalized using biofilms grown for seven days, as it was observed that at this time point, the biofilm biomass was comparable between the 29 °C and 37 °C/5 % CO₂ conditions (Fig. 4, Fig. 6). Highly resolved protein bands were detected and ranged from <20 kDa to >220 kDa (Fig. 9A) in replica biofilm preparations. Additionally, immunoblotting allowed for detection of specific proteins within each sample (Fig. 9B). Results suggest increased expression of GroEL (60 KDa), a member of the heat shock protein family, in the biofilm phenotype at 37 °C/5 % CO_2_ compared to biofilm at 29 °C (Fig. 9B).

## 4 Discussion

The genus *Leptospira* includes a diverse group of non-pathogenic and pathogenic species [10,[29], [30], [31], [32]] and leptospirosis transmission involves a complex epidemiological cycle with diverse animal hosts, persistence in the environment, as well as biofilm formation [12,15,33]. Despite the sensitivity of *Leptospira* spp. to harsh conditions encountered in changes to salinity, osmolarity, and pH, these spirochetes are widespread in bodies of water, sewage, and soil, and endure adverse environmental circumstances [[34], [35], [36], [37], [38]].

Pathogenic species of *Leptospira* form biofilms in the renal tubules of naturally infected Norway rats [15], which have body temperatures that range from 36 °C to 38 °C [39]. In Salvador, a city at the Northeast of Brazil, an urban environment where the average daily temperature is 25.7 °C ± 2.35 °C [40], a diversity of nonpathogenic leptospires were isolated from biofilms formed in surfaces bathed by open sewage [10]. In a rural region of India, a pathogenic *Leptospira* was isolated from biofilms formed in waterlogged paddy field soils where the daily temperature was 28 ± 1 °C [12,41]. The impact of temperature during the formation of biofilms by bacteria differs depending on the bacterial species involved. *In vitro* cultivation experiments with *Pseudomonas aeruginosa* revealed a notable temperature-dependent effect, where lower temperatures (20 °C) led to increased biofilm formation in comparison to higher temperatures (25 °C, 30 °C, and 37 °C) [42]. Conversely, the Gram-negative bacterium *Vibrio parahaemolyticus* formed significantly more biofilms at higher temperatures (15 °C and 37 °C) compared to those at lower temperatures (4 °C and 10 °C) [43]. Though *Leptospira* spp. exhibit an adaptability and ecological versatility to form biofilms, relatively little is known about the process, or the effect of temperature on their development.

The growth of leptospires *in vitro* is routinely performed using Ellinghausen McCullough media with modifications by Johnson and Harris (EMJH) at 28 to 30 °C [44]. Non-pathogenic leptospires thrive best within this temperature range and at lower temperatures, with cell lengths ranging from 6 to 20 μm, depending on the species, growth phase, nutrient availability, and temperature [34,44]. Similarly, the isolation of pathogenic *Leptospira* from animal and human hosts was also limited to growth with media at 28 to 30 °C [45]. However, the recent development of HAN media, which contains chemically defined components that includes DMEM/Ham's Nutrient mixture F12 and hemin as a source of iron, overcomes these limitations and supports isolation of *Leptospira* directly from host samples at both 28 to 30 °C and at 37 °C [22,31,46,47]. This in turn facilitates studies to elucidate how *Leptospira* modify gene and protein expression in response to environmental cues encountered during host infection to identify virulence factors expressed *in vivo* [22,[48], [49], [50]]. Given that *Leptospira* form biofilms *in vivo* during renal colonization of rats [15], we used *L*. *biflexa* as a model to investigate biofilm formation at temperatures encountered during host infection.

*L. biflexa* has the same growth rate whether cultured in HAN media at 37 °C/5 % CO_2_ or at 29 °C. Unexpectedly, increased cell length was detected in *L*. *biflexa* cultured at 37 °C/5 % CO_2_ compared to those at 29 °C and suggests an alteration in their growth metabolism at higher temperatures. Studies have shown that the pathogenic *L. borgpetersenii* serovar Hardjo strains JB197 and HB203 cultivated at 37 °C and 29 °C express different protein profiles between temperatures, including heat shock proteins with decreased expression at 29 °C [51].

Previous studies that investigated biofilm formation by leptospires *in vitro* are limited to their growth in EMJH or T80/40/LH at 29 °C–30 °C [[8], [9], [10], [11],^14^]. These biofilms are characterized by a maturation phase within two to four days that is quickly followed by detachment [8,10,11]. In HAN media, *L. biflexa* also formed biofilm at 29 °C but the maturation phase lasted until at least day 10, with detachment starting on day 11. Similarly, *L*. *biflexa* in HAN media formed biofilms at 37 °C/5 % CO_2_, and these were sustained beyond day 11 as confirmed by increased biomass detection using crystal violet.

*Leptospira* biofilm exopolymeric matrix is composed of proteins, eDNA and mucopolysaccharides [9,13,15]. In the present work, confocal microscopy analysis suggests that the biomass of biofilms formed by *L. biflexa* at both 29 °C and 37 °C/5 % CO_2_ in HAN media comprises more proteins than dsDNA. Additionally, a clearly defined layer of protein is delineated on the bottom of the *L. biflexa* biofilm matrix during all phases of formation and in both growth conditions which suggests they play a major role in cell to surface adhesion. Analyses of their 3D structure identified proteins between dsDNA (cells and eDNA) suggesting a role in biofilm structural support. The importance of extracellular DNA (eDNA) in maintaining the 3D structure in biofilms of *L*. *biflexa* has previously been demonstrated since enzymatic digestion of eDNA resulted in a significant disruption of the biofilm; yet it was still possible to observe trace amounts of biofilm after this treatment [13]. Based on our results, it is plausible to hypothesize a significant role for proteins in the establishment and adherence of biofilms to solid matrices, as well as their ability to persist.

Although crystal violet staining is the most widely used technique to semi-quantify biofilm biomass, it can sometimes show significant limitations, including poor reproducibility and sensitivity, and the potential risk of overestimating or underestimating biofilm biomass due to variations in the wash steps [52]. The semi-quantification of *Leptospira* biofilm biomass using crystal violet resulted in background levels of detection in negative controls, which showed elevated absorbance values. At certain time points, the CV assay indicated the absence of biofilm (after subtraction of background values) even though biofilm was clearly visible. Given these methodological limitations, we propose the combined use of CV with other techniques to effectively demonstrate biofilm formation by leptospires. These techniques include fluorescence microscopy using specific dyes, as well as scanning and transmission electronic microscopy [10,14,15,25]. Advantages of CLSM include the possibility of using specific dyes to stain the biofilm matrix components such as eDNA, proteins and exopolysaccharides [53]. This approach allows for a qualitative structural analysis of *Leptospira* biofilms, including the identification of component locations within the biofilm, the determination of biofilm composition, and the quantification of the biofilm biomass and other variables such as biovolume, surface area, and average thickness [9,10,25].

Ultrastructural analysis of *L. biflexa* biofilm at 29 °C and 37 °C/5 % CO_2_ in HAN media detected acidic exopolysaccharidic matrix as stained by ruthenium red. This component is also present in biofilms formed by pathogenic *Leptospira* in the renal tubules of Norway rats [15], as well as proteins and membrane vesicles-like structures. In Gram-negative bacteria, membrane vesicles-like structures can be produced in biofilms by blebbing of the outer membrane that in turn protect cells against antimicrobials [54]. The combination of eDNA and proteins (intracellular and cell surface associated) is observed in the biofilm matrix of clinical strains of *Clostridium difficile* [55], while in *Staphylococcus aureus*, this combination includes the possibility of exopolysaccharide [56], as observed in this work. Biofilm extracellular polymeric matrix components, including polysaccharides, proteins, extracellular DNA (eDNA), water, and others, participate in cell-to-cell adhesion and adhesion of biofilms to biotic or abiotic surfaces [5,57,58]. The biofilm matrix provides the infrastructure and can contain multifunctional membrane vesicles that are shed by the biofilm cells [54,58]. The proteins that compose the biofilm matrix include secreted, cytoplasmic, and outer membrane proteins (OMP). These molecules can participate in the initial attachment to surfaces and contribute to the structure and stability of the biofilms [59]. Flagella-like structures were also detected in biofilms by TEM (Fig. 8) though it is not clear if these are flagella associated with viable cells since the endoflagella of intact *Leptospira* are internal and confined to the periplasmic space [34].

Transcriptomics of *L.* *biflexa* in biofilm at 29^o^C compared to planktonic cells has previously demonstrated differential gene expression e.g., *ompL36* was overexpressed in late biofilm, while *grpE* was downregulated in mature biofilm [11], but validation of expression of proteins remains to be confirmed. Proteomic analysis of *Aggregatibacter actinomycetemcomitans* showed that several proteins are differentially expressed in biofilms, including GroEL and other OMPs, compared with planktonic cells [17]. Similarly, the proteomic profile of *Mycobacterium tuberculosis* showed that 64 % of proteins were more highly expressed in biofilms compared to planktonic cells, including GroEL, which had a high network connectivity with several other proteins and further highlighting its role during biofilm formation [60]. The proteomic data generated herein validates our methodologies to generate sufficient protein amounts for proteomic analyses, and our preliminary investigations suggest differential protein expression between the phenotypes tested in different growth conditions, and as illustrated for GroEL. Though the role for GroEL in biofilms of *Leptospira* is not yet known, our methods confirm that it is expressed. Additional proteomic studies of interest include 2-D gel electrophoresis and/or total protein quantification by liquid chromatography and mass spectroscopy [46,47,51]. With these techniques, it will be possible to identify proteins present in biofilms at different phases of development, compare levels of expression at different temperatures compared to planktonic cells [11], determine their role using genetically manipulated strains [26], and validate targets identified using *in silico* approaches related to c-di-GMP turnover and signaling [21].

The current work presents new insights into the structure and composition of biofilm formation by *L. biflexa*. These non-pathogenic model leptospires grow in HAN media not only at 29 °C, but also at 37 °C/5 % CO_2_, a temperature encountered during persistent host infection and/or warm climate environments. *L. biflexa* formed biofilms under both conditions tested, with biofilms of higher biomass being formed at 37 °C/5 % CO_2_ compared to 29 °C. The detection of more proteins than dsDNA in the biofilm matrix, and as a distinctive layer on the bottom of the biofilm, suggests a significant role for proteins in attachment to abiotic surfaces. The use of sterile glass slides for biofilm formation overcomes limitations in alternative methods such as microtiter plates with EMJH media [9,14] to provide sufficient proteins amounts for comprehensive proteomic analysis. Our findings elucidate mechanisms underlying biofilm formation by leptospires and models that can facilitate in-depth proteomic analysis to understand their role during biofilm formation.

## CRediT authorship contribution statement

**Priscyla dos Santos Ribeiro:** Writing – review & editing, Writing – original draft, Visualization, Supervision, Methodology, Investigation, Formal analysis, Data curation, Conceptualization. **Judith Stasko:** Writing – review & editing, Writing – original draft, Investigation, Formal analysis. **Adrienne Shircliff:** Writing – review & editing, Writing – original draft, Methodology, Investigation, Formal analysis. **Luis Guilherme Fernandes:** Writing – review & editing, Writing – original draft, Methodology, Investigation, Formal analysis. **Ellie J. Putz:** Writing – review & editing, Writing – original draft, Methodology, Investigation, Formal analysis. **Claire Andreasen:** Writing – review & editing, Writing – original draft, Supervision, Methodology, Investigation. **Vasco Azevedo:** Writing – review & editing, Writing – original draft, Supervision, Methodology, Investigation, Conceptualization. **Paula Ristow:** Writing – review & editing, Writing – original draft, Visualization, Supervision, Methodology, Investigation, Data curation, Conceptualization. **Jarlath E. Nally:** Writing – review & editing, Writing – original draft, Visualization, Supervision, Methodology, Investigation, Data curation, Conceptualization.

## Declaration of competing interest

The authors declare that they have no known competing financial interests or personal relationships that could have appeared to influence the work reported in this paper.

## Data Availability

Data will be made available on request.
